# Contribution of Age, Brain Region, Mood Disorder Pathology, and Interindividual Factors on the Methylome of Human Microglia

**DOI:** 10.1016/j.biopsych.2021.10.020

**Published:** 2021-10-30

**Authors:** Lot D. de Witte, Zhaoyu Wang, Gijsje L.J.L. Snijders, Natalia Mendelev, Qingkun Liu, Marjolein A.M. Sneeboer, Marco P.M. Boks, Yongchao Ge, Fatemeh Haghighi

**Affiliations:** Mental Illness Research, Education and Clinical Center, James J Peters VA Medical Center, Bronx; Department of Psychiatry, Icahn School of Medicine at Mount Sinai, New York, New York; Mental Illness Research, Education and Clinical Center, James J Peters VA Medical Center, Bronx; Nash Family Department of Neuroscience, Icahn School of Medicine at Mount Sinai, New York, New York; Mental Illness Research, Education and Clinical Center, James J Peters VA Medical Center, Bronx; Department of Psychiatry, Icahn School of Medicine at Mount Sinai, New York, New York; Mental Illness Research, Education and Clinical Center, James J Peters VA Medical Center, Bronx; Nash Family Department of Neuroscience, Icahn School of Medicine at Mount Sinai, New York, New York; Mental Illness Research, Education and Clinical Center, James J Peters VA Medical Center, Bronx; Nash Family Department of Neuroscience, Icahn School of Medicine at Mount Sinai, New York, New York; Department of Psychiatry, Icahn School of Medicine at Mount Sinai, New York, New York; Department of Psychiatry, University Medical Center Utrecht, UMC Utrecht Brain Center, Utrecht University, Utrecht, the Netherlands; Department of Translational Neuroscience, University Medical Center Utrecht, UMC Utrecht Brain Center, Utrecht University, Utrecht, the Netherlands; Department of Psychiatry, University Medical Center Utrecht, UMC Utrecht Brain Center, Utrecht University, Utrecht, the Netherlands; Department of Neurology, Icahn School of Medicine at Mount Sinai, New York, New York; Mental Illness Research, Education and Clinical Center, James J Peters VA Medical Center, Bronx; Department of Psychiatry, Icahn School of Medicine at Mount Sinai, New York, New York; Nash Family Department of Neuroscience, Icahn School of Medicine at Mount Sinai, New York, New York

## Abstract

**BACKGROUND:**

Transcriptome studies have revealed age-, disease-, and region-associated microglial phenotypes reflecting changes in microglial function during development, aging, central nervous system homeostasis, and pathology. The molecular mechanisms that contribute to these transcriptomic changes are largely unknown. The aim of this study was to characterize the DNA methylation landscape of human microglia and the factors that contribute to variations in the microglia methylome. We hypothesized that both age and brain region would have a large impact on DNA methylation in microglia.

**METHODS:**

Microglia from postmortem brain tissue of four different brain regions of 22 donors, encompassing 1 patient with schizophrenia, 13 patients with mood disorder pathology, and 8 control subjects, were isolated and assayed using a genome-wide methylation array.

**RESULTS:**

We found that human microglial cells have a methylation profile distinct from bulk brain tissue and neurons, and age explained a considerable part of the variation. Additionally, we showed that interindividual factors had a much larger effect on the methylation landscape of microglia than brain region, which was also seen at the transcriptome level. In our exploratory analysis, we found various differentially methylated regions that were related to disease status (mood disorder vs. control). This included differentially methylated regions that are linked to gene expression in microglia, as well as to myeloid cell function or neuropsychiatric disorders.

**CONCLUSIONS:**

Although based on relatively small samples, these findings suggest that the methylation profile of microglia is responsive to interindividual variations and thereby plays an important role in the heterogeneity of microglia observed at the transcriptome level.

Microglia are the resident population of immune cells of the brain parenchyma. They are derived from myeloid progenitors in the yolk sac and populate the brain early during embryo-genesis (1). In response to environmental insults, microglia are involved in initiating and regulating inflammatory responses in the central nervous system. They have also emerged as key players in neurodevelopment and neuronal functioning in adulthood (2). Microglia dysfunction has therefore been hypothesized to play a critical role in neurodevelopmental disorders, as well as in neurodegenerative and neuropsychiatric disorders (3,4), motivating increasing research into the role of microglia in health and disease.

Thus far, most studies have analyzed microglial phenotypes and changes at the level of gene expression. Using genome-wide transcriptome analyses and targeted quantitative polymerase chain reaction, it was shown that microglia show brain region–specific phenotypes (5-7). This was also observed for rodent and human microglia at the protein level (6,8). The microglial transcriptome has further been shown to be influenced by sex and aging (9-14). Disease-associated transcriptome alterations have been previously reported in microglia isolated from postmortem brain tissue of patients with Alzheimer’s disease (15-18) and multiple sclerosis (19,20) and corresponding animal models (21,22). Taken together, these studies suggest that microglia form a heterogeneous population of cells across brain regions, sexes, age ranges, and diseases. It is thought that these are reflections of intrinsic properties of different subtypes of microglia with specialized functions in health and pathology (22-25).

The molecular mechanisms that contribute to the heterogeneity of microglia that is observed at the transcriptome level are not yet clear. Epigenetic mechanisms are important regulators of gene expression, with DNA methylation being the most stable and long-lasting epigenetic mechanism of gene regulation (25-27). DNA methylation modifications are transmitted to daughter cells and therefore are important for providing a specific and stable profile of gene expression in different tissues and their specific cell types. Most of these DNA methylation patterns are generated during development. In addition, adjustments in DNA methylation patterns occur in response to specific molecular triggers, which allows the cell to adapt gene expression to a dynamic environment. Mutations and variants in genes that regulate DNA methylation have been associated with several psychiatric disorders (27). In addition, DNA methylation of blood and bulk brain tissue has been related to psychiatric pathology and the response of patients with psychiatric disorders to medication (28-32).

The aim of this study was to analyze the DNA methylation landscape of human microglia and factors that contribute to variation in microglia DNA methylation profiles. Based on previous studies, we hypothesized that age and brain region are important drivers of the variance in human microglia DNA methylation patterns. We isolated microglia from the medial frontal gyrus (MFG), superior temporal gyrus (STG), sub-ventricular zone (SVZ), and thalamus (THA) and performed a genome-wide methylation array. By including microglia isolated from four different brain regions with a wide spectrum of ages from donors with and without psychiatric disorders, we were able to analyze the contribution of region, age, sex, and disease status on the microglia methylome. As an exploratory analysis, we also investigated DNA methylation patterns between microglia isolated from cases with mood disorders and nonpsychiatric control subjects. We further analyzed RNA sequencing (RNA-seq) data of 50 microglia samples that are also in the methylation dataset to investigate how DNA methylation variation and mood disorder related–changes are related to gene expression levels.

## METHODS AND MATERIALS

### Donors

Fresh postmortem brain tissue from the MFG, STG, SVZ, and THA was obtained from the Netherlands Brain Bank (http://www.brainbank.nl). Permission to collect human brain material was obtained from the Medical Ethical Committee of the VU University Medical Centre, Amsterdam, The Netherlands. All donors had provided informed consent for the use of brain tissue and clinical information for research purposes prior to death. Demographic and clinical characteristics of the donors are summarized in Table 1 and described more extensively in Table S1 in Supplement 2. The microglia samples that were used in this study were isolated as part of a larger initiative to characterize microglia across different ages and brain regions, as well as in relation to genotype (33). We included different brain regions because there is evidence for a heterogeneous phenotype of microglia across regions, especially for white versus gray matter, cortical versus subcortical regions, and cerebellum versus cortical regions (5,8,20,34-36). The brain regions of interest in this study, the MFG, STG, SVZ, and THA, were selected based on these previous studies, the fact that they have been related to affect circuits in mood disorders before (37,38), as well as possibilities to obtain larger pieces of fresh brain tissue from the Netherlands Brain Bank.

### Human Primary Microglia Isolation

Because microglia are only a smaller subpopulation (2%–8%) of cells in the human brain (39,40), most of the methylation signals derived from microglia are probably lost when performing bulk brain tissue analysis of DNA methylation. We therefore used human primary microglia isolated from fresh postmortem brain tissue as described previously (41-43). Briefly, fresh postmortem brain tissue from these four brain regions was mechanically and enzymatically dissociated in a glucose-potassium-sodium buffer (8.0 g/L NaCl, 0.4 g/L KCl, 1.77 g/L Na_2_HPO_4_ · 2H_2_O, 0.69 g/L NaH_2_PO_4_ · H_2_O, 2.0 g/L D-(1)-glucose, 0.3% bovine serum albumin [Merck]; pH 7.4) and supplemented with collagenase type I (3700 units/mL; Worthington Biochemical) and DNase I (200 μg/mL; Roche) at 37 °C for 60 minutes while shaking. Because the composition of SVZ is different from the other regions with more white matter, this tissue was digested using an alternative protocol of 0.2% trypsin (Invitrogen) for 30 minutes while shaking. A Percoll (Amersham, Merck) gradient was generated to separate the microglia from myelin and cellular debris. The middle layer enriched for microglia was washed twice, and microglia were pulled down with CD11b-conjugated magnetic beads (Miltenyi Biotec). Microglia were either lysed using 200-μL RLT buffer (Qiagen) for RNA-seq analysis or centrifuged and the cell pellet stored for DNA methylation analyses.

### DNA Methylation

DNA was extracted with QIAamp DNA Micro Kit (Qiagen). Genomic DNA was bisulfite converted (Zymo Research), and CpG methylation was determined using Illumina Infinium EPIC Human Methylation BeadChip microarrays, as described previously (44). Data and quality control (QC) analyses were performed using R language 4.0.3 (45), an environment for statistical computing, and Bioconductor 2.13 (46). Raw data files (.idat) were processed by minfi package (47). For all samples assayed, >97% probes passed detection call rates (*p* < .00005) (Figure S1A in Supplement 1). Sex QC analysis did not identify any samples to be mislabeled relative to reported sex (Figure S1B in Supplement 1). For QC sample tracking of different brain regions, we used the 59-single nucleotide polymorphism probes included in the EPIC 850 bead array, confirming that individuals with multiple sample specimens across multiple brain regions grouped together (Figure S1C in Supplement 1).

Multidimensional scaling (MDS) was used for visualizing clustering of the DNA methylation data across various factors (i.e., age, brain regions, diagnosis). DNA methylation levels in beta values were transformed to M-values (logit transformation of beta values) and used in subsequent analyses. To quantify the contributions of different factors to overall DNA methylation levels, we performed principal component analysis (PCA) on the M-value matrix, which was linear adjusted for age and sex, and selected the top 20 principal components (PCs), which accounted for >99.9% of total variability within the methylation data. For every PC, we used analysis of variance to quantify the variance contributed by each factor. Percent variance reported for each factor was derived from the ratio of variances for each PC divided by the total variance summed across the 20 PCs. To identify differentially methylated regions (DMRs) between cases with mood disorder diagnosis and controls, we applied a linear mixed model with donor as random effect and age, sex, and brain region as fixed effect covariates using the dream method (48) as implemented in the variancePartition package (49) implemented in R. We obtained *t* statistics and associated *p* values for each CpG site. For each brain region separately, we also performed diagnostic comparisons using a linear model with sex and age as covariates. Quantile-quantile plots were generated for each model tested to check for *p* value inflation. We performed *p* value correction by multiplying these with lambda if necessary (Figures S2 and S3 in Supplement 1). The adjusted pointwise *p* values were then used for the identification of DMRs using the combined *p* values (comb-p) tool (50). Significant DMRs using Sidak correction (51) for multiple testing correction were reported.

To determine how the methylation profiles of microglia compare with other neural cell types, we combined publicly available EPIC 850K methylation data (52) (accession number GSE111165) from bulk brain tissue, as well as isolated neuronal (NeuN+) and glial (NeuN−) cells with our microglia dataset; applied surrogate variable analysis on the M-value matrix using R package surrogate variable analysis to adjust for batch effects; and visualized the sample clustering via MDS.

### RNA Sequencing

Among the 52 microglia samples from subjects with mood disorders, 50 have been sequenced via RNA-seq as part of our microglia genomics atlas initiative (33). Total RNA was extracted with the RNeasy mini kit (Qiagen) in combination with an RNase-Free DNase Set (Qiagen) for additional DNA removal, according to manufacturer’s protocol. RNA library preparations and sequencing reactions were conducted at GENEWIZ. SMART-Seq v.4 Ultra Low Input Kit for Sequencing was used for full-length complementary DNA synthesis and amplification (Clontech), and Illumina Nextera XT library was used for sequencing library preparation. Briefly, complementary DNA was fragmented and adapter was added using transposase, followed by limited-cycle polymerase chain reaction to enrich and add index to the complementary DNA fragments. The final library was assessed with Agilent TapeStation. The sequencing libraries were multiplexed and clustered on a flowcell. After clustering, the flowcell was loaded on the Illumina HiSeq instrument according to manufacturer’s instructions. The samples were sequenced using a 2 × 150 paired end configuration. Image analysis and base calling were conducted by HiSeq Control Software. Raw sequence data (.bcl files) generated from Illumina HiSeq were converted into fastq files and demultiplexed using Illumina’s bcl2fastq 2.17 software. RNA-seq data were processed using the RAPiD pipeline (53). RAPiD aligns samples to the hg38 genome build using STAR (54) (v.2.7.2a) using the GENCODE v.30 transcriptome reference and calculates QC metrics using Picard (55). RNA-seq QC was performed by applying filters to remove samples meeting the following criteria: 1) samples with <10 million reads aligned from STAR, 2) samples with >20% of the reads aligned to ribosomal regions, and 3) samples with <10% of the reads mapping to coding regions.

The voomWithDreamWeights function in the R package variancePartition was used to get log counts per million (CPM) matrix, where the design matrix consists of age, sex, diagnostic status, and brain region as fixed effect and donor as random effect. Based on the mean-variance trend plot of logCPM generated from the voomWithDreamWeights function (48), we empirically chose the threshold of 1 to filter out undetected genes (defined as mean logCPM ≥ 1) in both diagnostic groups. To quantify contributions of different factors toward variation of microglia gene expression profiles, analyses were performed similarly as described for DNA methylation data above. Differential gene expression analysis was run parallel to methylation analysis with the same model settings.

## RESULTS

**Figure 1.**
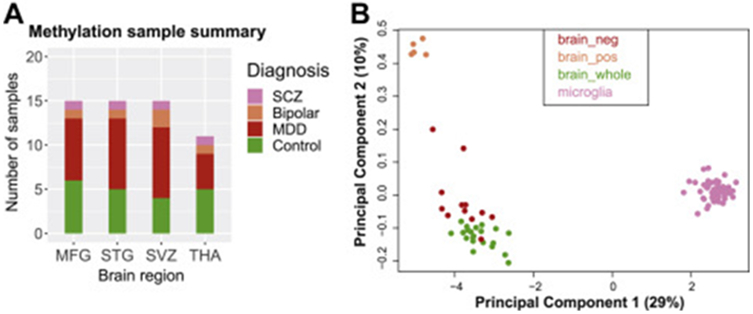
**(A)** Sample distribution across disease status and brain region used in DNA methylation assays. **(B)** Multidimensional scaling plot to visualize clustering of microglia compared with previously published DNA methylation data from bulk brain, neuron, and glia samples (accession number GSE111165) using the Illumina HumanMethylation EPIC platform. Samples from different datasets were first combined and adjusted for batch effect using surrogate variable analysis and subsequently used to generate a multidimensional scaling plot. MDD, major depressive disorder; MFG, medial frontal gyrus; SCZ, schizophrenia; STG, superior temporal gyrus; SVZ, subventricular zone; THA, thalamus.

We used the EPIC 850K array to characterize DNA methylation profiles of microglia isolated from a total of 56 sample specimens obtained from 22 individuals across four different brain regions. Individual cases included patients with major depressive disorder (MDD), bipolar disorder, or schizophrenia, as well as individuals without any neurologic or neuropsychiatric disorder (Table 1; Table S1 in Supplement 2). The distribution of the samples across disorders and regions is depicted in Figure 1A. Using a publicly available methylation dataset, we showed that microglia display a distinct methylation profile compared with bulk brain tissue, isolated neurons, and mixed glial populations (Figure 1B).

### Influence of Age and Sex on Microglia Methylome

**Figure 2.**
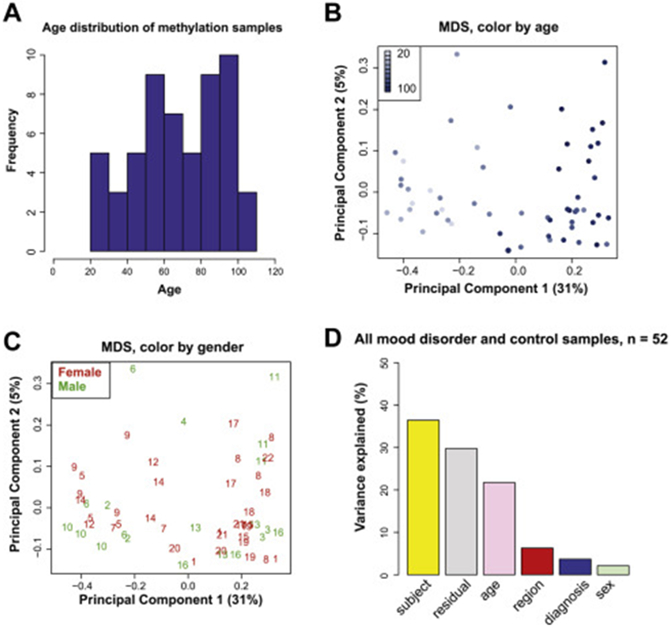
**(A)** Sample age distribution. **(B)** MDS plot labeled by age and **(C)** MDS plot labeled by sex, using DNA methylation data from autosomal chromosomes only. **(D)** Bar plot showing contribution of different demographic factors, brain region, and psychiatric diagnosis to observed DNA methylation variability in top 20 principal components. MDS, multidimensional scaling.

The ages of the samples ranged from 21 to 103 years (Table S1 in Supplement 2), as shown in the age distribution plot in Figure 2A. As expected, we found that microglia DNA methylation patterns track with chronological age (Figure 2B). In addition, as expected, sex chromosomes show distinct DNA methylation profiles, wherein individual cases separated based on reported sex (Figure S1B in Supplement 1), but following removal of sex chromosomes, no such sex separation was observed within autosomal chromosomes (Figure 2C). To quantify the contribution of age and sex on DNA methylation patterns, we performed PCA, and in line with previous studies in other cell types and tissues (52), we found that age has a much larger impact on the microglial methylome, explaining 21.7% of the variability, than sex, with only 2.1% (Figure 2D; Figure S4 in Supplement 1). However, the individual subject effect appeared to be strongest, explaining 36.5% of the variability in the DNA methylation data.

### Influence of Region, Psychiatric Pathology, and Individual on Microglia Methylome

**Figure 3.**
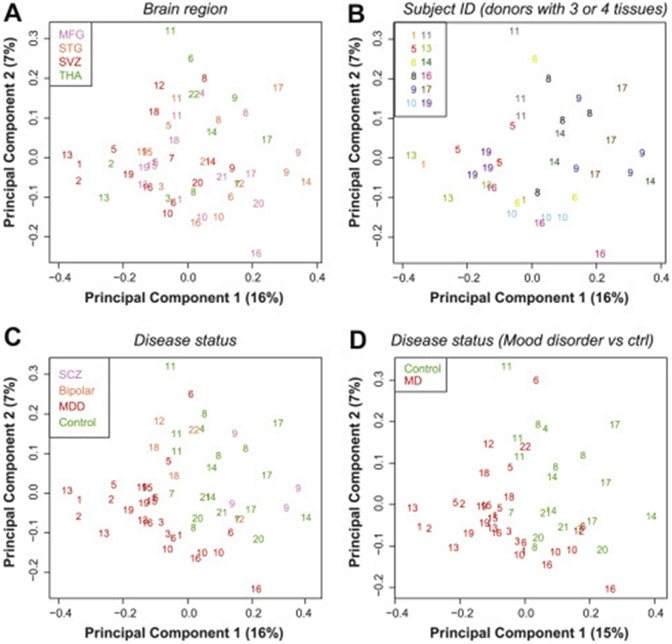
Multidimensional scaling plots of DNA methylation data adjusted for age and sex. **(A)** Brain region. **(B)** Only individuals for whom we included three or four regions from the same individual, colored by individual ID. **(C)** Disease status, including MDD, bipolar disorder, and SCZ. **(D)** MD, including MDD and bipolar cases and excluding SCZ. Ctrl, control; ID, identification; MD, mood disorder; MDD, major depressive disorder; MFG, medial frontal gyrus; SCZ, schizophrenia; STG, superior temporal gyrus; SVZ, subventricular zone; THA, thalamus.

**Figure 4.**
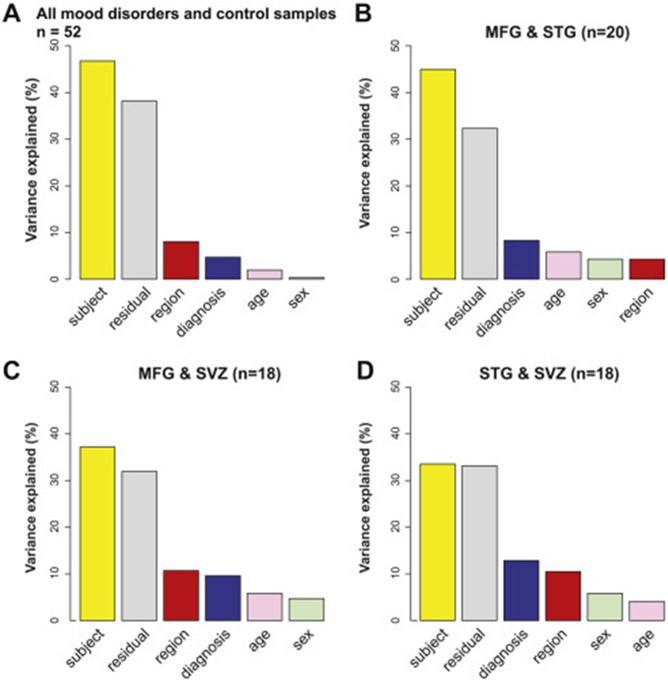
**(A)** Bar plot showing contribution of different factors to DNA methylation variability (M-value matrix was first linear adjusted for age and sex) in top 20 principal components using all samples. **(B–D)** Bar plots using paired samples for which DNA methylation data are available for two brain regions: MFG and STG **(B)**, MFG and SVZ **(C)**, and STG and SVZ **(D)**, with all analyses adjusted for age and sex. MFG, medial frontal gyrus; STG, superior temporal gyrus; SVZ, subventricular zone.

To investigate the influence of these and other factors on the microglial methylome, in subsequent analytic models, we included age and sex as covariates. Visualizing the DNA methylation data by brain region via an MDS plot (Figure 3A), we showed that the samples did not cluster by region but clustered by individual (Figure 3B). Specifically, DNA methylation patterns from different brain regions from the same individual often clustered together (Figure 3A, B). We also visualized the DNA methylation data via MDS by diagnosis status, and we showed that control subjects tend to cluster separately from patients with either bipolar disorder or MDD, but not schizophrenia (Figure 3C). This is further supported by grouping bipolar disorder and MDD together in a mood disorder group in Figure 3D. To quantify the contributions of these factors including brain region, diagnosis and other individual-dependent variables, we performed PCA on all samples, as well as on paired microglia samples from two different regions. It should be noted that we adjusted DNA methylation levels (M-values) for age and sex using a linear model, performed PCA on the adjusted M-values, and calculated the percentage of variance contributed by each variable in the top 20 PCs. In all analyses, we found that individual-dependent variables accounted for the largest variance components, ranging from 33.6% to 46.8% (Figure 4A-D). Brain region and diagnosis explained between 4.7% and 12.9% of the variance (Figure 4A-D). While we controlled for age and sex effects, residual effects were detected.

### Influence of Region, Diagnosis, and Individual on the Transcriptome of Microglia

**Figure 5.**
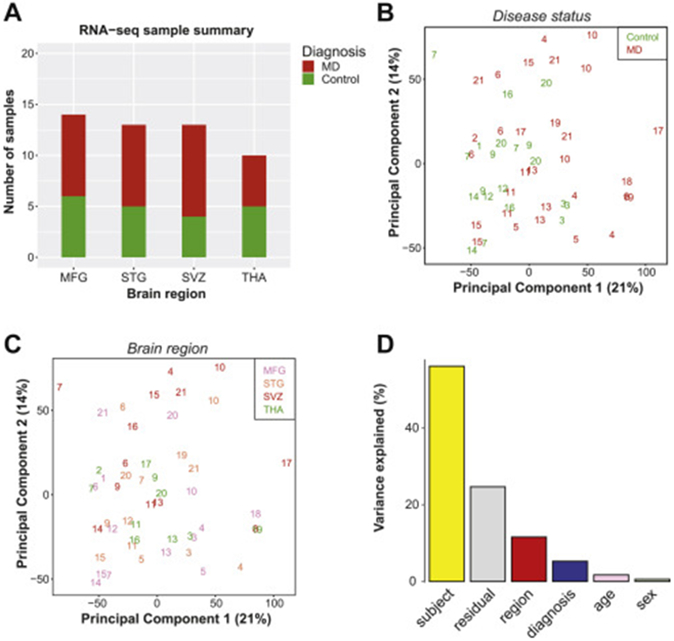
**(A)** Sample distribution across disease status (control or MD) and brain region used in RNA-seq (*n* = 50). **(B, C)** Principal component analysis plot of top two principal components labeled by MD diagnosis **(B)** and brain region **(C)**. **(D)** Bar plot of contribution of different factors to observed RNA transcript variability (logCPM matrix was first linear adjusted for age and sex) in top 20 principal components in RNA-seq data. MD, mood disorder; MFG, medial frontal gyrus; RNA-seq, RNA sequencing; STG, superior temporal gyrus; SVZ, subventricular zone; THA, thalamus.

For 50 of the 52 microglial samples used for methylation, we also had transcriptome data available. The distribution of these samples is shown in Figure 5A. Visualization of these data via MDS plots shows no separation by disease status or brain region (Figure 5B, C). Similar to the methylome data, we found that RNA transcript data from the same individual tend to cluster together. This is quantified via PCA, which shows that the influence of individual-dependent variables is also larger than diagnostic state or brain region (Figure 5D).

### DMRs in Microglia From Patients With Mood Disorder

Because diagnosis state explained between 4.7% and 12.9% of the variance of the microglial methylome, we performed an exploratory analysis to identify DMRs between patients with mood disorder and control subjects. We first analyzed DMRs using all available samples, accounting for sex, age, and brain region as covariates in the statistical models. Secondarily, these analyses were also performed for the MFG, STG, and SVZ brain regions separately. We did not analyze THA samples separately because of the low sample size and a skewed quantile-quantile plot. Results are summarized in Table 2, results for all DMRs identified are provided in Tables S2 and S3 in Supplement 2, and results for selected DMRs of interest are plotted in Figures S5 and S6 in Supplement 1. DNA methylation regulates gene expression and is generally associated with gene repression but can also have other effects, including enhancement of gene expression (56). By using our RNA-seq data, we therefore explored whether the identified DMRs are related to expression of the annotated gene (Tables S2 and S3 in Supplement 2). Finally, for the across-tissue analysis, we compared methylation and gene expression differences between the control and mood disorder groups in an exploratory fashion for the 36 DMRs for which we detected gene expression in microglia. After we filtered genes expressed in microglia with mean logCPM ≥ 1 for each group, we found that genes annotated to 5 of 36 DMRs were significantly differentially expressed (pointwise *p* values < .05), including *DST*, *MSLN*, *UNC119B*, *CD79B*, and *HRH1*. A correlation of *r* = −0.1668 (*p* = .3381) was found between methylation and gene expression levels (Figure S7 in Supplement 1). For promoter DMRs only (*n* = 12), inverse correlation with gene expression was greater (*r* = −0.5531, *p* = .0622). The results for the *HRH1* locus are highlighted in Figure S8 in Supplement 1.

## DISCUSSION

The aim of this study was to investigate the DNA methylation landscape of human microglia and factors that contribute to variation of the methylation landscape. As expected, we found that human microglial cells have a distinct methylation profile that is influenced by age. Furthermore, by isolating cells from different brain regions of the same donors, we further showed that interindividual factors had a much larger effect on the methylation landscape of microglia than regional differences. In exploratory analyses, comparing patients with mood disorder versus control subjects, we found a number of DMRs of microglial-expressed genes that were associated with mood disorder.

Prior studies across multiple tissues and cell types have shown that aging has a major impact on DNA methylation (57,58). In fact, DNA methylation has been the most promising molecular biomarker of the aging processes (57,59). Additionally, the microglia transcriptome has also been shown to be influenced by aging (6,7,10,13,60). Therefore, it is not surprising that data from this study also showed a clear association between microglia DNA methylation with age. These age-related DNA methylation changes might play an important role in age-related disorders because increasing evidence points to a causal role of microglia in neurodegenerative diseases, such as Alzheimer’s disease (61-63).

In disagreement with our hypothesis, we did not find a major impact of brain region on the microglia methylome, although this analysis was limited by the fact that samples of a variable number of regions were available for the different donors. Microglial samples from different regions of the same individuals did not separate by region, but rather by individual. These data suggest that interindividual factors have a much higher impact on DNA methylation of microglial cells than brain region. These data are in line with a recent study of Rizzardi *et al.* (64) showing that the methylation profile of neuronal but not non-neuronal cells, which include microglial cells, differ across brain regions. In contrast to our methylation data in human microglia, rodent studies have shown clear region-specific microglia heterogeneity (5,36). Such cross-species differences between human and animal studies (6,65) may be attributed to the nature of experimental animal models. Animals have the same genetic background, are also largely exposed to the same environment (i.e., same rearing conditions, same food, same pathogens), and have the same cause of death with samples collected all in the same batch. In our human cohort, these factors vary largely among individuals but are the same within one individual. We hypothesize that genetic background, medical history, cause of death, and other lifestyle factors all may contribute to the observed variability in the methylation profile of microglia between different donors, which limits the possibility to detect regional-specific effects. Microglia are seen as the sentinels of the central nervous system, which rapidly respond to changes in the microenvironment over the life span, and our data suggest that these may be more pronounced than potential brain region–specific differences in DNA methylation.

In our exploratory analysis, we identified various DMRs that are correlated with mood disorder status. These include DMRs annotated to several genes of interest, because the genes are highly expressed in microglia and related to microglial immune functions or neuropsychiatric disorders. Among these genes is *HRH1*. We found a DMR in the promoter region of this gene, and gene expression was significantly upregulated in mood disorder cases. This gene encodes the histamine receptor H1, which has been shown to be involved in microglia activation (66,67). Other genes of interest include *SLC29A3*, *SPHK2*, *PDK2*, and *TRADD*, which are all highly expressed in microglia and known to modulate immune functions of myeloid cells including endosomal lysosomal function and inflammatory responses (68-74). Additionally, we found DMRs annotated to several genes previously associated with psychiatric disorders including *ARID1B, ADCY9*, and *DIP2A* (75-77), as well as *MCF2L*, and homeobox genes including *HOXA3, HOXA4, HOXA5, HOXB7*, and *MEIS1*, which have been reported to be associated with pathology of Alzheimer’s disease (16,78). In our regional analysis, we identified a DMR within the promoter of a circadian clock gene, *PER3*. *PER3* is recognized as a core component of the circadian rhythm system that regulates various physiological functions. Dysregulation of the clock genes has been linked to both bipolar disorder and MDD (78-81).

This study has several limitations. As a human postmortem study, the sample size is necessarily small. The technical challenges for microglia isolation with the requisite access to fresh autopsy brain tissue further compounded the limited sample size. Nevertheless, with a total of 56 brain tissue specimens, this represents the largest human microglia DNA profiling study to date. While for such a small case-control study, we did not expect to find significant methylation differences by diagnostic state, we did find clustering of sample individuals by mood disorder diagnosis relative to nonpsychiatric control subjects. In addition, although we did identify significant DMRs in patients with mood disorder versus control subjects, whether these are associated with mood disorder diagnosis or related to confounders associated with disease status, such as cause of death or medication, could not be determined with this sample size. These potential covariates could only be retrieved through retrospective chart review, and with the limited information and limited sample size, we could not control for these factors in our analysis; therefore, these findings warrant replication in future studies.

In conclusion, findings from this study suggest that human microglia respond to changes in their microenvironment via transcriptional regulatory mechanisms and specifically DNA methylation alterations. Because environmental exposures contribute significantly to the risk of brain disorders, data from this study are important in furthering our understanding of the role of microglial cells in this process and support further research toward understanding how the patterns of DNA methylation of microglia are affected by changes in environment and how this may translate to changes in microglia function.

## Supplementary Material

Supplementary Table 1

Supplementary Figures

## Figures and Tables

**Table 1.** Demographics by Psychiatric Diagnoses for All Brain Donors

Demographics	Total, N = 22	Control Subjects, n = 8	Major Depressive Disorder, n = 10	Bipolar Disorder, n = 3	Schizophrenia, n=1
Regions, No. of Donors					
Medial frontal gyrus	16	6	8	1	1
Superior temporal gyrus	14	5	7	1	1
Subventricular zone	15	4	8	2	1
Thalamus	11	5	4	1	1
Age, Years, Mean ± SD *(p* Value)^a^	68.4 ± 23.3	79.1 ± 17.1	59.6 ± 26.1 (.33)	73.3 ± 25.1 (.044)	55
Sex, Female/Male (p Value)^b^	14/8	6/2	6/4 (.50)	3/0 (.48)	1/0
Postmortem Delay, Minutes, Mean ± SD (p Value)	454 ± 116	450 ± 104	469 ± 125 (.37)	458 ± 152 (.46)	335

a p Value compared with control subjects (f test).

b p Value compared with control subjects (χ^2^ test).

**Table 2.** Differentially Methylated Regions Related to Mood Disorder Status

Group/Type of DMR	Cross-Tissue Analysis	Medial Frontal Gyrus	Superior Temporal Gyrus	Subventricular Zone
Control Subjects, No. of Samples	20 (derived from 8 donors)	6	5	4
Mood Disorder, No. of Samples	32 (derived from 13 donors)	8	9	10
DMR < .05 FDR, *n*	81	30	71	24
DMR Mapping to Promoter Regions of Protein-Coding Genes, *n*	33	7	27	11
DMR Mapping to Genes Expressed in Microglia, Mean Log CPM > 1, *n*	36	13	32	10
DMRs Mapping to Genes With Potential Gene Expression Differences With a Liberal Pointwise p Value < .1, *n*	8 (*DST, MSLN, UNC119B, CD79B, HRH1, ARID1B, HDAC9, FAM214A)*	0	4 (*FAM53A, TRIM14, SPNS2, TRIOBP)*	1 *(MEIS1)*
Other Genes of Interest	PDK2, SPHK2, SLC29A3, TIAM2, HOXA3, HOXA4, HOXA5, HOXB7, ADCY9, TRADD	PER3, FIGNL1	LGALS8, TRAK1	HLADPA1, DDB2, FGF20

CPM, counts per million; DMR, differentially methylated region; FDR, false discovery rate.

**Table:** KEY RESOURCES TABLE

Resource Type	Specific Reagent or Resource	Source or Reference	Identifiers	Additional Information
Add additional rows as needed for each resource type	Include species and sex when applicable.	Include name of manufacturer, company, repository, individual, or research lab. Include PMID or DOI for references; use “this paper” if new.	Include catalog numbers, stock numbers, database IDs or accession numbers, and/or RRIDs. RRIDs are highly encouraged; search for RRIDs at https://scicrunch.org/resources.	Include any additional information or notes if necessary.
Antibody				
Bacterial or Viral Strain				
Biological Sample	Genomic DNA and RNA	microglia isolated from post-mortem human brain	GSE182360	
Cell Line				
Chemical Compound or Drug				
Commercial Assay Or Kit				
Deposited Data; Public Database				
Genetic Reagent				
Organism/Strain				
Peptide, Recombinant Protein				
Recombinant DNA				
Sequence-Based Reagent				
Software; Algorithm				
Transfected Construct				
Other				
