# Supplementary material for: Contribution of Age, Brain Region, Mood Disorder Pathology, and Interindividual Factors on the Methylome of Human Microglia
Source: Biol Psychiatry. Author manuscript; Available in PMC 2024 Jun 17. (PMC11181298; doi:10.1016/j.biopsych.2021.10.020)
Supplement: Supplementary Figures [file NIHMS1995917-supplement-Supplementary_Figures.pdf]

Contribution of Age, Brain Region, Mood Disorder Pathology, and Interindividual Factors on the Methylome of Human Microglia

Supplement 1

Supplementary figure 1.

A

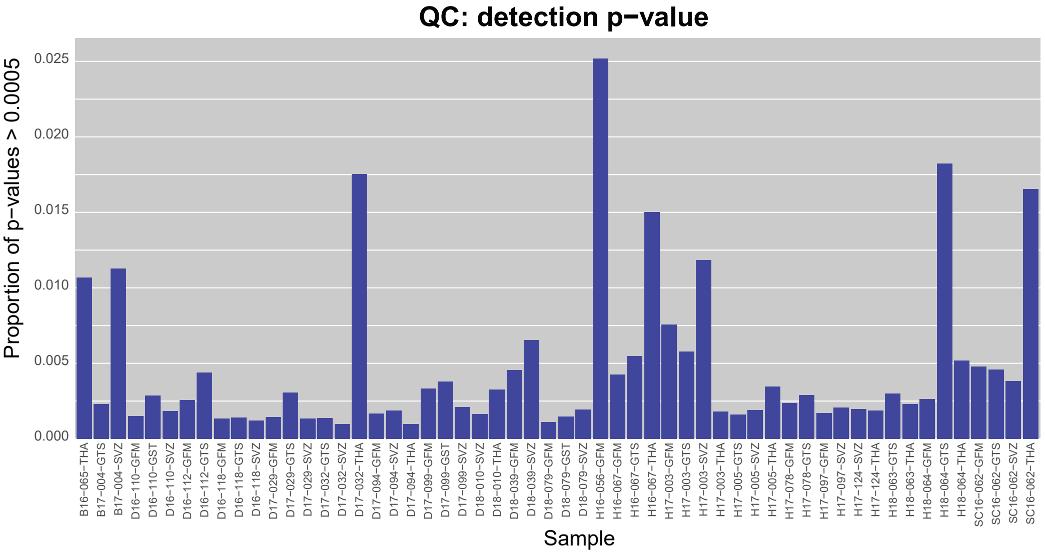

B

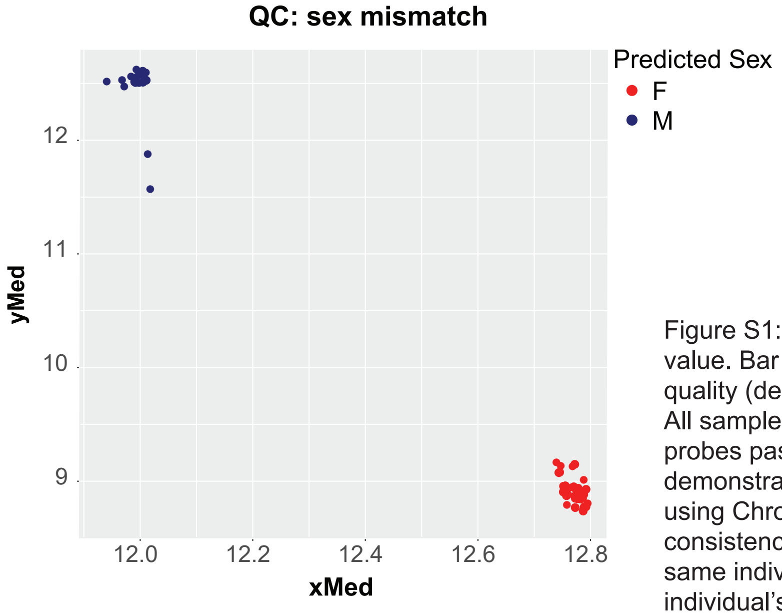

C

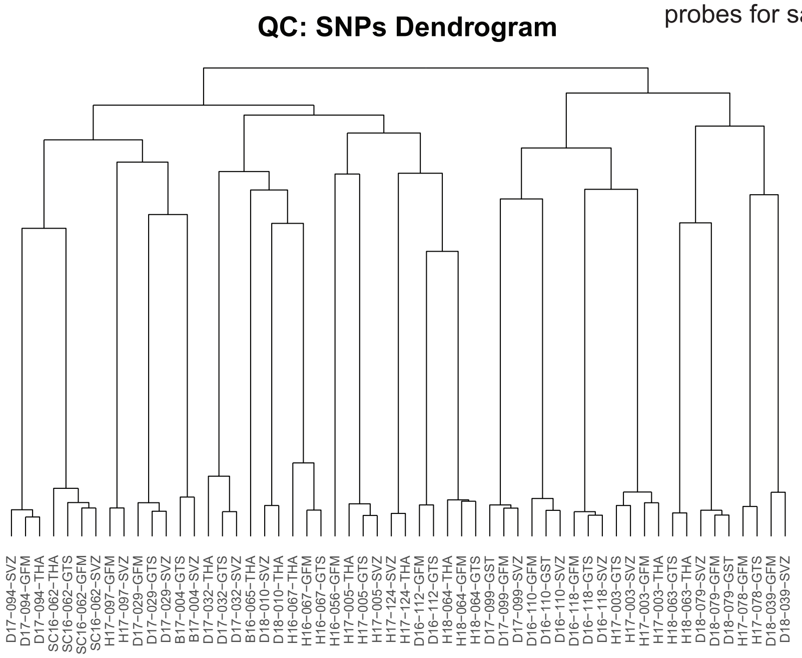

Figure S1: Methylation data quality control. (A) Detection p-value. Bar graph showing the proportion of probes with poor quality (detect p>0.0005) probes for each biological sample. All samples had high quality data, with at least 99% of probes passing criteria; (B) Sexprediction. Plot demonstrates consistency for predicted and reported sex, using Chromosome X and Y median intensity; (C) Genotype consistency check with samples from different regions of same individual. The dendrogram shows clustering of each individual’s samples by genotypes derived from the 59 SNP probes for samples. The y-axis shows Euclidean distance.

Supplementary figure 2

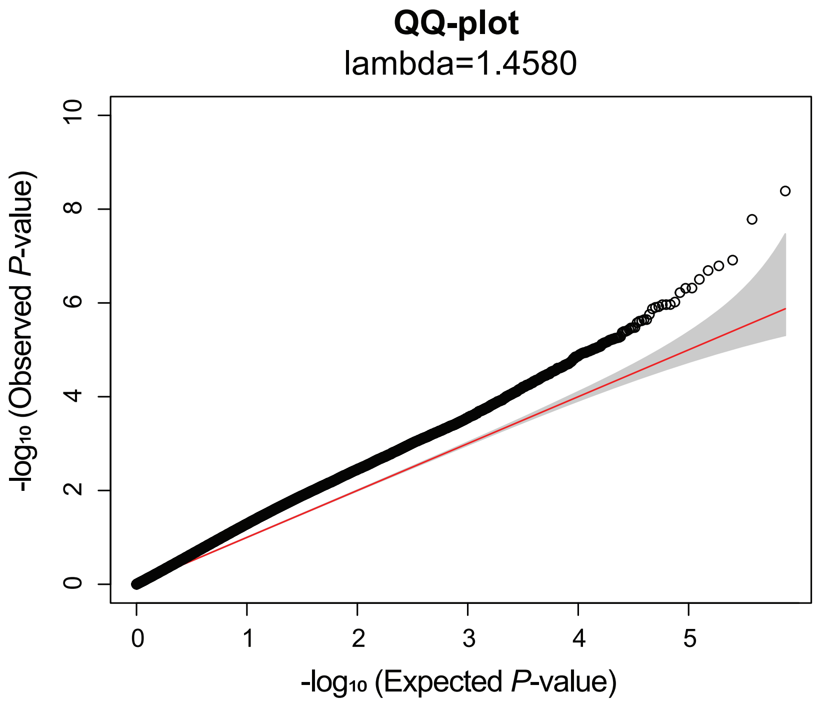

Figure S2: QQ plot of p-values of comparison between Mood disorder and control in the model including all sample specimens. The calculated Lambda = 1.458, which was used to adjust for Type-1 Error inflation in the cross-tissue analysis.

Supplementary figure 3

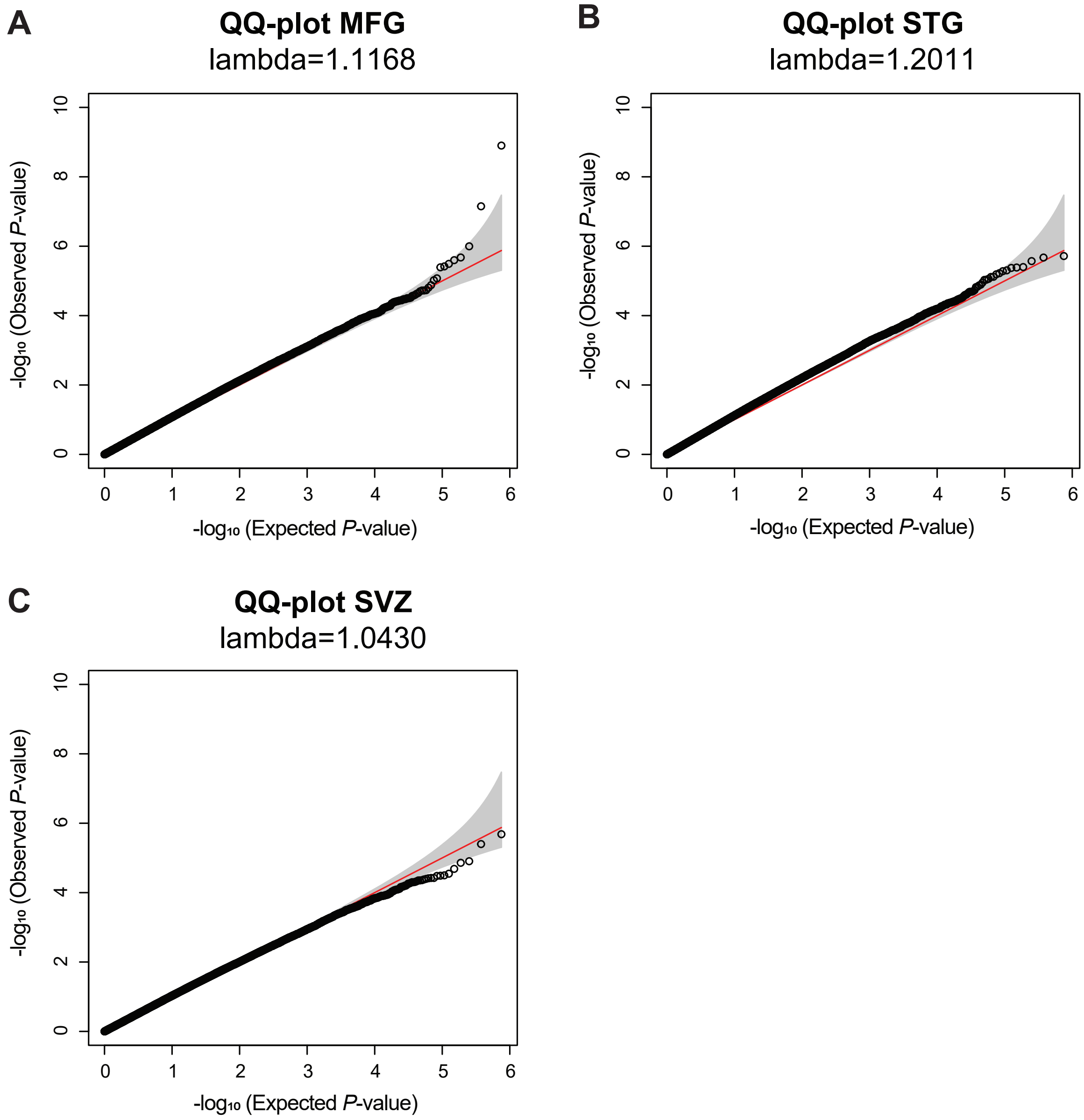

Figure S3: QQ plots of p-values of comparison between Mood disorder and control in the region- specific models. Lambdas for MFG, STG and SVZ are acceptable.

Supplementary figure 4

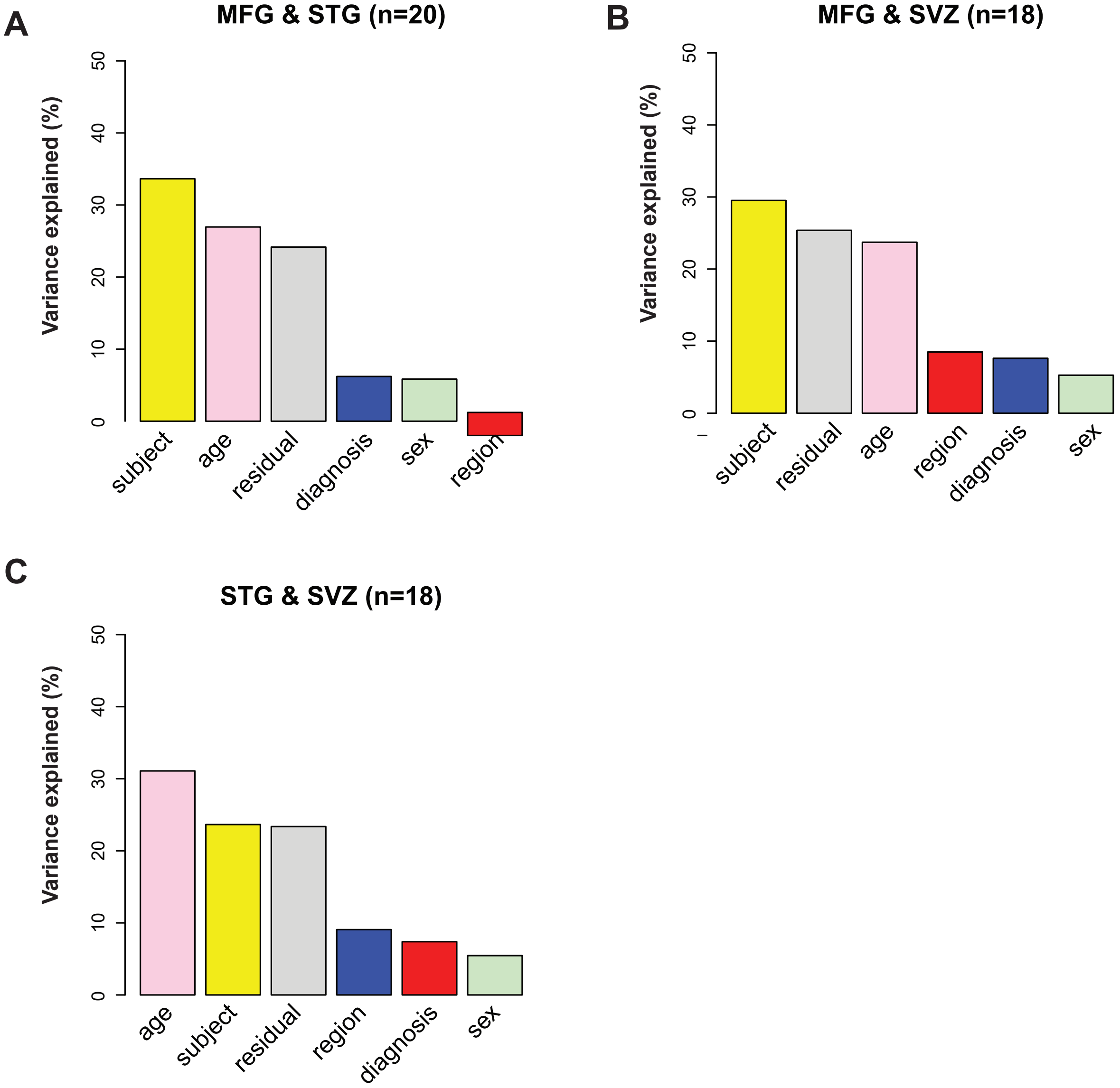

Figures S4: Bar plot showing contribution of different factors to DNA methylation variability (M- value matrix was not adjusted for age and sex) in top 20 principal components, using paired samples for which DNA methylation data is available for two brain regions (i.e., MFG & STG, MFG & SVZ, and STG & SVZ respectively).

Supplementary figure 5

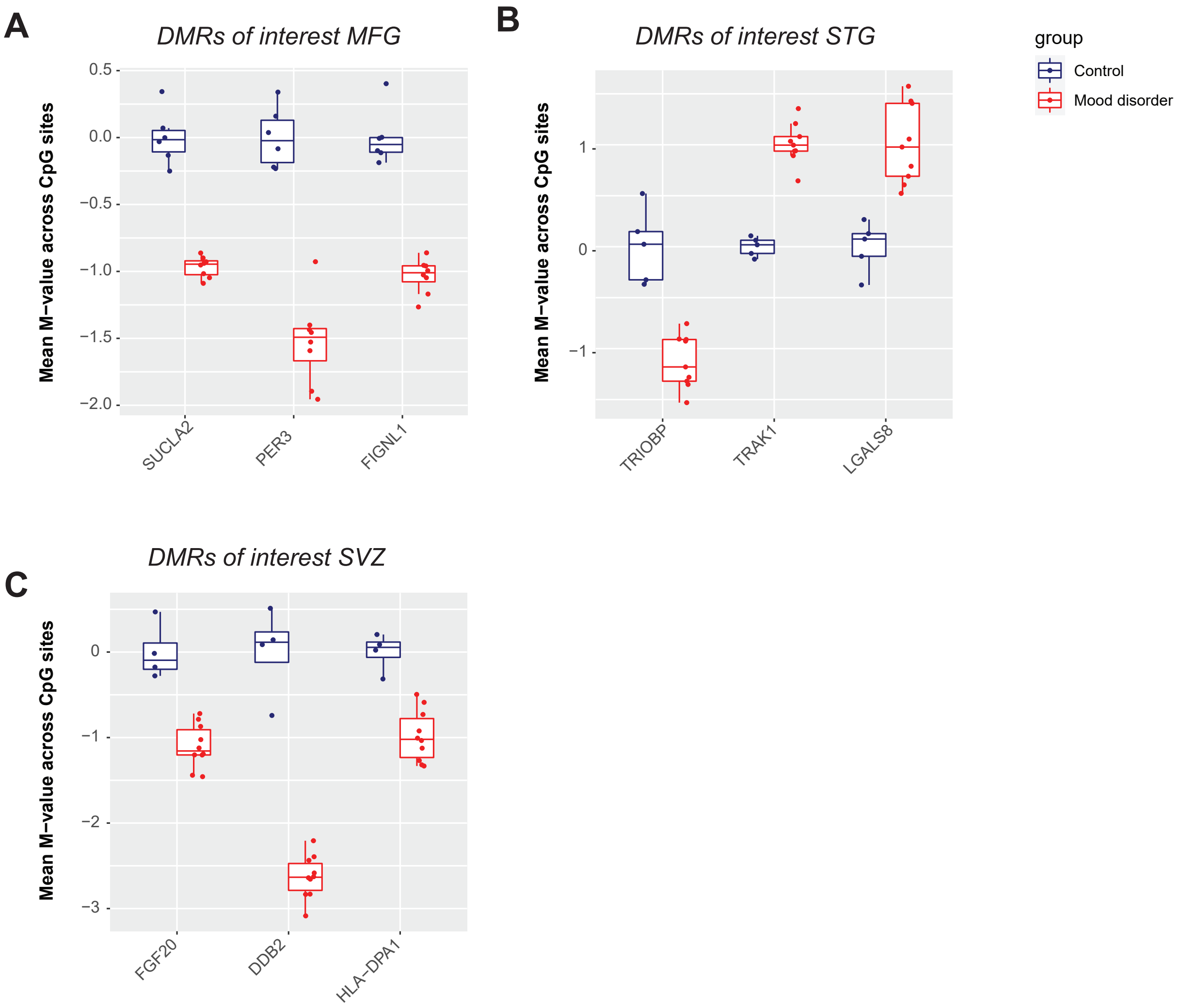

Figure S5: Examples of differentially methylated regions (DMRs) that were found significant after correcting for multiple testing in the cross-region analysis comparing individuals diagnosed with combined in mood disorders6 and controls, showing their mean methylation M-values across CpG sites on the y-axis, with control groups in blue and mood disorder in red.

Supplementary figure 6

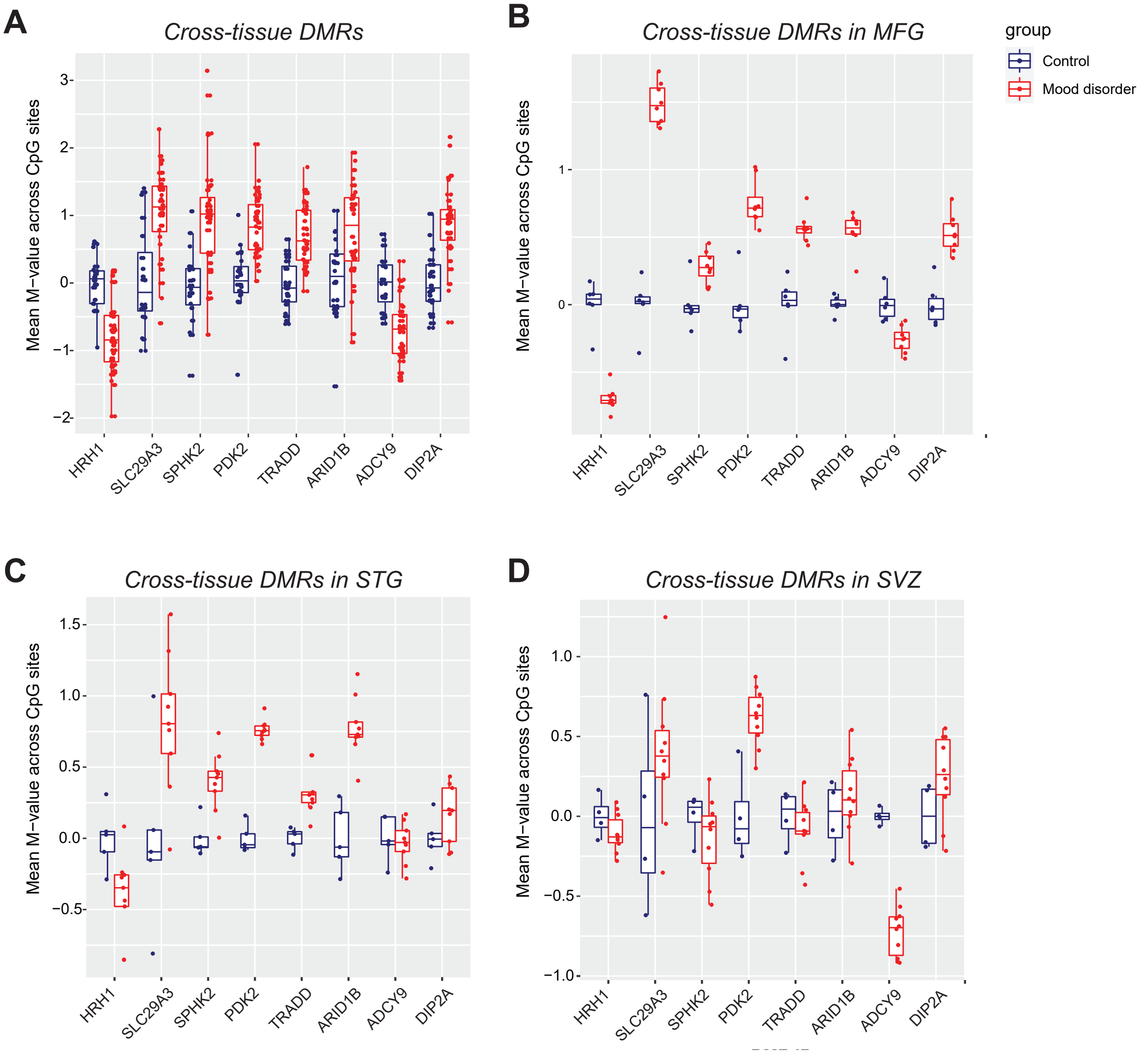

Figure S6: Region-specific differentially methylated regions (DMRs) that were found in comparative analysis, showing control groups in blue and mood disorder in red, with mean methylation M-values across CpG sites on the y-axis.

Supplementary figure 7

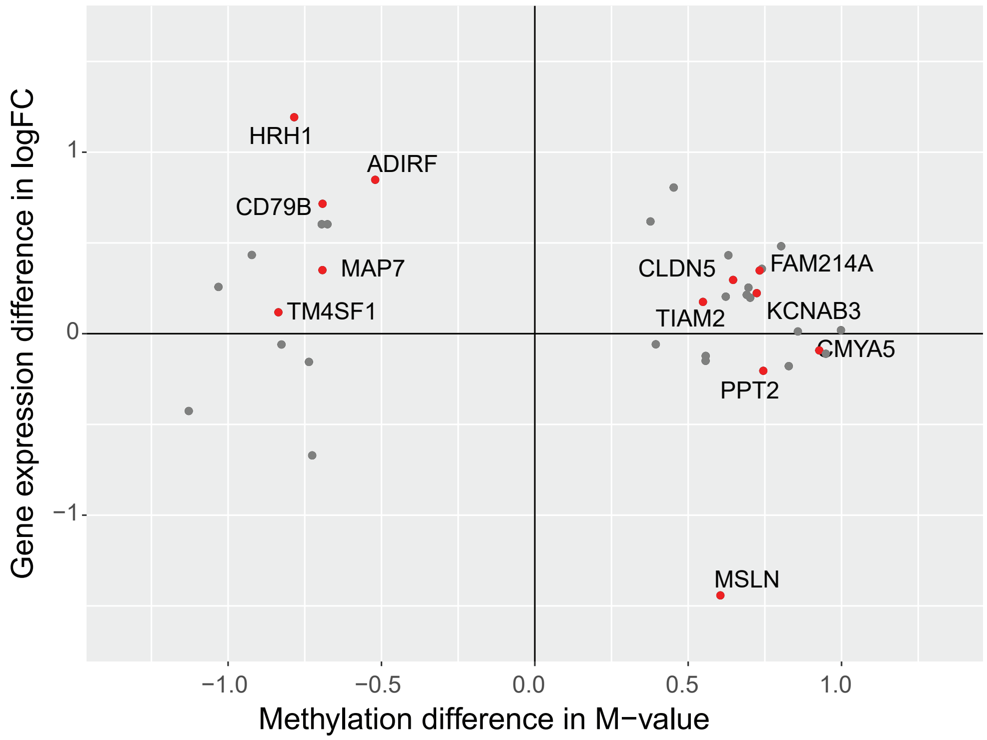

Figure S7: Scatter plot of methylation difference (in M-value) on x-axis and gene expression difference (in logFC) on y-axis of 35 DMRs with associated expressed genes. Promoter DMRs are highlighted in red and labelled with the annotated gene names.

Supplementary figure 8

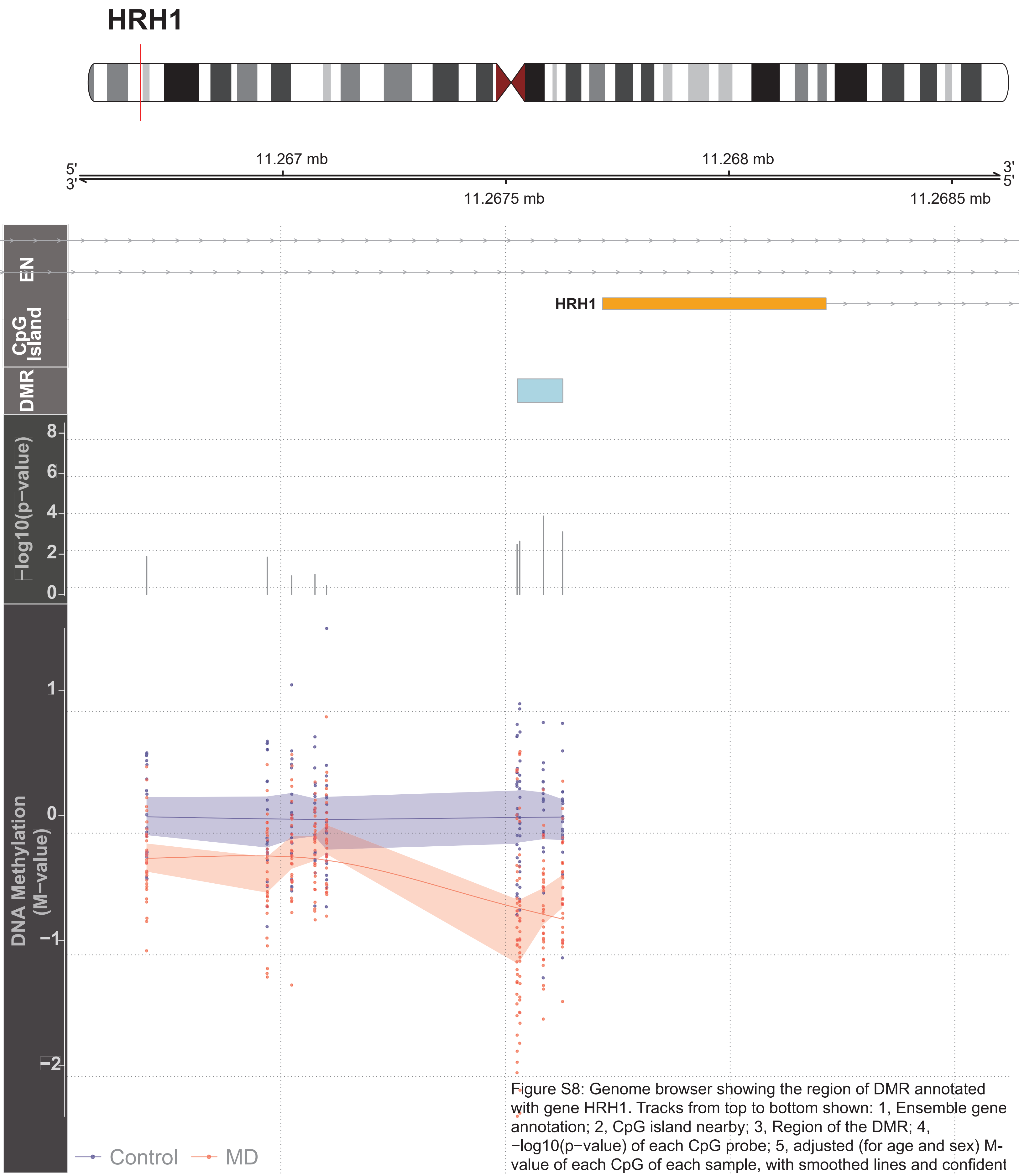

Figure S8: Genome browser showing the region of DMR annotated with gene HRH1. Tracks from top to bottom shown: 1, Ensemble gene annotation; 2, CpG island nearby; 3, Region of the DMR; 4, −log10(p−value) of each CpG probe; 5, adjusted (for age and sex) M-value of each CpG of each sample, with smoothed lines and confident interval, points are coloured into two diagnosis groups

Data Access:

DNA Methylation data is available on GEO (Gene Expression Omnibus) with accession number GSE191200 and RNA-seq data is available with accession number ng00105.
